# Evaluation of clinical outcomes of implants placed into the maxillary sinus with a perforated sinus membrane: a retrospective study

**DOI:** 10.1186/s40902-016-0097-6

**Published:** 2016-12-05

**Authors:** Gwang-Seok Kim, Jae-Wang Lee, Jong-Hyon Chong, Jeong Joon Han, Seunggon Jung, Min-Suk Kook, Hong-Ju Park, Sun-Youl Ryu, Hee-Kyun Oh

**Affiliations:** Department of Oral and Maxillofacial Surgery, School of Dentistry, Chonnam National University, 77, Yongbong-ro, Buk-gu, Gwangju, Republic of Korea

**Keywords:** Perforation of sinus membrane, Resorbable collagen membrane, Survival rate of implant, Perforation repair, Complications after bone graft

## Abstract

**Background:**

The aim of this study was to evaluate the clinical outcomes of implants that were placed within the maxillary sinus that has a perforated sinus membrane by the lateral window approach.

**Methods:**

We examined the medical records of the patients who had implants placed within the maxillary sinus that has a perforated sinus membrane by the lateral approach at the Department of Oral and Maxillofacial Surgery of Chonnam National University Dental Hospital from January 2009 to December 2015. There were 41 patients (male:female = 28:13). The mean age of patients was 57.2 ± 7.2 years at the time of operation (range, 20–76 years). The mean follow-up duration was 2.1 years (range, 0.5–5 years) after implant placement. Regarding the method of sinus elevation, only the lateral approach was included in this study.

**Results:**

Ninety-nine implants were placed in 41 patients whose sinus membranes were perforated during lateral approach. The perforated sinus membranes were repaired with a resorbable collagen membrane. Simultaneous implant placements with sinus bone grafting were performed in 37 patients, whereas delayed placements were done in four patients. The average residual bone height was 3.4 ± 2.0 mm in cases of simultaneous implant placement and 0.6 ± 0.9 mm in cases of delayed placement. Maxillary bone graft with implant placement, performed on the patients with a perforated maxillary sinus membrane did not fail, and the cumulative implant survival rate was 100%.

**Conclusions:**

In patients with perforations of the sinus mucosa, sinus elevation and implant placement are possible regardless of the location and size of membrane perforation. Repair using resorbable collagen membrane is a predictable and reliable technique.

## Background

Maxillary sinus elevation, also known as maxillary sinus lift, is regarded as a standardized technique for the recovery of the masticatory function followed by placing implants in the atrophic maxillary posterior region. Elevation of the Schneiderian membrane in the maxillary sinus is a very delicate procedure; thus, perforation of the mucosa during an operation occurs frequently (10–55%) [1–5]. Perforation of the membrane makes a direct communication to the maxillary sinus, and via this communication the graft material can be scattered into the sinus space; however, it also can be the cause of infection or sinusitis. Vlassis et al. [6] reported that sinus perforation occurs during the process of osteotomy when forming the window rather than the process of separating the mucosa from the bony wall. They also reported that smokers show a higher perforation rate than non-smokers and other factors, such as antral septa and narrow sinus, can lead to perforation of the maxillary sinus. Though anatomical factors can be overcome by the operator, predisposing causes towards the sinus perforation, such as smoking, is important for prevention. In addition, history of previous maxillary sinus operation can lead to a large perforation [4, 7]. Ardekian et al. [8] reported 85% perforation rate of sinus membranes with a residual ridge of 3 mm, while 25% perforation rate for residual ridge of 6 mm.

Small perforations (<5 mm) at the site of folded mucosa are reported to be healed by itself [4, 9]. However, if the perforation is large (≥5 mm) and located on an unfavorable site such as the middle third of the lower marginal area of the lateral window, the perforation requires repair by a collagen membrane [2, 4, 7, 8, 10, 11], fibrin adhesive [12], block graft [6, 9], or suturing using resorbable material [12], in order to prevent the loss of graft material. If the perforation cannot be blocked, the procedure should be discontinued.

If the perforated sinus membrane is treated properly, there is no adverse effect on the survival of the implant placed in a perforated maxillary sinus [7]. However, few studies have investigated the clinical outcomes and survival rate of these procedures. The aim of this study was to evaluate the clinical outcomes of implants, which were placed after sinus elevation of a perforated maxillary sinus membrane.

## Methods

### Patients

This retrospective study reviewed the medical records of cases of maxillary sinus perforations that occurred during a sinus lift procedure at the Department of Oral and Maxillofacial Surgery, Chonnam National University Dental Hospital from January 2009 to December 2015. All surgical procedures were done by one oral and maxillofacial surgeon. This study included 41 patients (male:female = 28:13; mean age, 57.2 ± 7.2 years; age range, 20–76 years). Mean follow-up period was 2.1 years (range, 0.5–5 years) after implant placement. The study excluded patients who went to other dental clinics for prosthetic restoration and were absent from follow-up appointment without notice. Maxillary sinus elevation was performed using the lateral approach. Perforation of the mucosa was immediately documented in the medical records.

As the clinical setting of the repair for the perforated membrane, if the size of the perforation was more than 5 mm or the graft material was expected to be scattered via the perforation, the repair was performed. Implants were placed simultaneously with the sinus floor augmentation if the residual bone height was >2 mm, and initial stability of the implant was observed. Initial stability was confirmed clinically by the surgeon based on the mobility of the implant fixture. Otherwise, the implant was placed more than 4 months (maturation period of the graft material) after the sinus floor augmentation. There were no immediately loaded implants.

In this study, we evaluated the following parameters: (1) distribution of age and gender; (2) types of graft materials for maxillary sinus bone graft; (3) timing of implant placement by residual bone height; (4) barrier membrane used for the repair of perforated sinus; (5) types of implant surfaces; (6) complications after bone graft; (7) timing of loading after implant placement; and (8) survival rates.

### Surgical technique

**Fig. 1:**
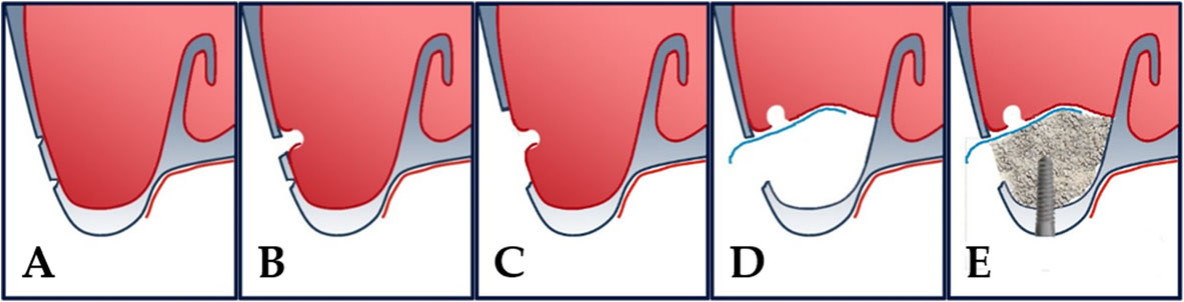
Schematic drawing of sinus perforation repair using an absorbable membrane. **a** Procedure of making the lateral window. **b** Occurrence of perforation. **c** Widening of the window. **d** Repair of the perforated membrane with an absorbable collagen membrane. **e** Bone graft in the elevated sinus and simultaneous implant placement

**Fig. 2:**
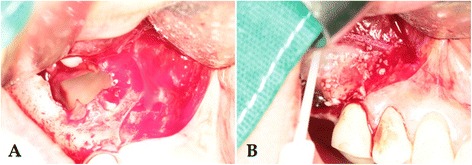
Intraoperative photographs. **a** Confirmation of the perforation in the sinus membrane. **b** Repair using collagen tape and packing of the graft material with fibrin adhesive

When the perforation of the maxillary sinus membrane was encountered during any case with a lateral approach, the bony window was widened with a Kerrison rongeur to expose the perforated sinus membrane after which the membrane was separated from sinus wall and elevated carefully (Figs. 1 and 2). After complete elevation of the perforated sinus membrane, the implant placement sites were prepared according to the protocol of the implant manufacturer while protecting the torn membrane. The autogenous bone, such as the maxillary tuberosity or ramus, was harvested and morcellized. The particulated bone was usually mixed with allograft or xenograft, and the fibrin sealant was injected onto the mixture of bone grafts.

The perforated membrane was repaired with the collagen membrane and fibrin sealant; bone graft materials were packed into the sinus under the collagen tape and implants were simultaneously placed into the prepared sites. Subsequently, the cover screws were connected. If primary stability could not be obtained, the implant placement was delayed approximately 4 months after sinus elevation. Sometimes, additional bone grafts were performed through the window of the lateral wall. The incised wound was sutured with 3-0 Mersilk^®^ (Ethicon Inc., Somerville, NJ, USA).

### Postoperative management

All patients were administered antibiotics (Augmentin^®^ - Ilsung pharmaceuticals Co., Seoul, Korea) thrice a day for 3 days after the surgery. In case of postoperative infection or maxillary sinusitis, antibiotics were prescribed for one to two additional weeks. The patients were given instructions to avoid elevating air pressure on the maxillary sinus; such as, “do not blow your nose” or “to sneeze with your mouth open,” for 2 weeks.

After surgery, all patients underwent periapical and panoramic radiography. There was no immediate loading on the implants. Implant prostheses were usually restored after 6 months (mean = 6.5 months; range: 5 to 12 months).

## Results

### Distribution of age and gender

A total of 99 implants were placed in 41 patients (28 men and 13 women). The ages ranged from 20 to 76 years (mean age, 57.2 ± 10.5 years).

### Types of graft materials for maxillary sinus bone graft

Mandibular ramus (19 patients) and maxillary tuberosity (13 patients) were used alone or in combination with other graft materials. Bio-Oss^®^ (Geistlich Pharma AG^®^, Wolhusen, Switzerland) was used as the xenograft in 16 patients. Ora-graft^®^ (DFDB - LifeNet Health^®^, Virginia Beach, VA, USA) was used as the allograft in 9 patients, and Novosis^®^ (CGBio^®^, Seongnam, Korea) was used as the alloplast in 1 patient (Table 1).

**Table 1:** Types of graft materials for the maxillary sinus graft

Graft materials	Number of patients
Ilium only	1
Ilium + xenograft	2
Ramus only	8
Ramus + xenograft	8
Ramus + allograft	1
Ramus + tuberosity + allograft	1
Tuberosity only	6
Tuberosity + allograft	4
Tuberosity + xenograft	2
Xenograft only	4
Allograft only	3
Alloplast only	1
Total	41

### Timing of implant placement by residual bone height

The preoperative dental CT images showed that the average residual bone height was 3.2 ± 1.9 mm (range, 0–9.7 mm), 3.4 ± 2.0 mm (range, 0–9.7 mm) in cases of simultaneous implant placement, and 0.6 ± 0.9 mm (range, 0–2.2 mm) in delayed implant placement. Of the 99 implants placed, 7 delayed implants were performed in 4 patients with alveolar crest bone heights <3 mm. The remaining implants were placed simultaneously during the sinus elevation (Table 2).

**Table 2:** Timing of implant placement by residual bone height

Residual bone height (mm)	Simultaneous (no. of implants)	Delay (no. of implants)
>7	3	0
7–3	40	0
<3	49	7
Total	92	7

### Barrier membranes used to repair the perforated sinus

Four types of resorbing collagen membranes were used for repair of the perforated sinus membrane; Rapiderm tape^®^ (Dalimtissen^®^, Seoul, Korea) in 23 patients, Ossguide^®^ (Osstem^®^, Seoul, Korea) in 12 patients, Collatape^®^ (Zimmer Biomet^®^, Warsaw, Indiana, USA) in 3 patients, and Geistlich Bio-Gide^®^ (Geistlich Pharma AG^®^, Wolhusen, Switzerland) in 3 patients.

### Types of implant surfaces

In this study, 57 resorbable blasting media (RBM) implants (52 Osstem^®^, Osstem Co., Seoul, Korea, 5 Megagen^®^, Megagen Co., Gyeongsan, Korea), 39 sand blasted with alumina and acid-etching (SA) implants (Osstem^®^, Osstem Co., Seoul, Korea), and 3 sand-blasted and acid etched (S&E) implants (Luna^®^, Shinhung Co., Seoul, Korea) were used. Regarding the timing of implant placement, 92 implants were placed simultaneously with sinus elevation in 37 patients and 7 implants were placed in 4 patients with delayed implantation 3~4 months after sinus augmentation (Table 3).

**Table 3:** Types of implant surface

Surface types	Simultaneous placement (no. of implants)	Delayed placement (no. of implants)	Total (%)
RBM	51	6	57 (57.6)
S&E	3	0	3 (3)
SA	38	1	39 (39.4)
Total	92	7	99 (100)

*RBM* resorbable blasting media, *S&E* sandblast and acid etched, *SA* sand blasted with alumina and acid-etching

### Complications after bone graft

The common postoperative complications of a maxillary sinus lift and implant placement includes edema and pain at the operation site and the time needed for relief from the complications. Among 41 patients, 14 patients (34.1%) were prescribed additional antibiotics due to postoperative infection or maxillary sinusitis. Eight patients had mild sinusitis based on radiographic findings (Waters’ view) and were prescribed metronidazole together with amoxicillin/clavulanic acid for an additional week. Infection symptoms (e.g., pain, swelling, localized fever on operative region) were observed in the other six patients without radiographic sign. In these cases, we prescribed amoxicillin/clavulanic acid for an additional week. Nasal congestion or rhinorrhea was present in 10 patients who were prescribed pseudoephedrine (Sudafed^®^, Johnson & Johnson Co., USA) for a week.

### Timing of loading after implant placement

There was no immediate loading on the implants. Implant loading was performed at an average 6.5 months after implant placement (range, 5–12 months).

### Survival rates

Patients with implant placement in the perforated sinus membrane showed no failure of implant. All implants included in the study showed 100% survival.

## Discussion

Hernandez-Alfaro et al. [13] classified perforations into three types depending on the size of perforation. Fugazzotto and Vlassis [10] classified them all into four types, including three types based on the location with two subtypes in type II. According to the data from previous studies [2, 4, 8–10], small perforations can self-repair. These studies suggest treatment based the size and position of the perforation. Many studies [6, 10, 14] suggest that perforation of the sinus can be treated with a resorbable membrane. Tiziano et al. [15] suggested that bioabsorbable membranes can be used to repair large perforations; in addition, they emphasized stabilization of the collagen membrane to repair perforated sinus membrane. In our study, we achieved successful surgical outcome after bone graft using resorbable collagen membrane and fibrin adhesive.

Bravetti et al. [16] reported that in elevation of the sinus membrane and insertion of bone graft or any other graft material, the Schneiderian membrane might be disrupted, and the graft material can be a source of chronic infection and sinusitis. On the other hand, Jensen et al. [5] reported that postoperative complications including infection and oro-antral fistula increased when a xenograft was used as the graft material. In this study, various materials used as a graft showed no impact on implant survival.

Some studies [17, 18] suggested that the implant with higher residual bone height (>5 mm) could have better primary stability and applicability for sinus elevation with simultaneous implant placement. Delayed implant placement was recommended for residual bone height of lower than 5 mm. In this study, in cases of simultaneous implant placement, the residual bone height was an average of 3.4 ± 2.0 mm. In cases of delayed implant placement group, residual bone height was 0.6 ± 0.9 mm on average. It may imply that primary stability is more important than residual bone height for determining the timing of implant placement.

Previous studies have reported acute maxillary sinusitis after sinus elevation of up to 26% [1, 19, 20]. Some studies recommend prophylactic antibiotics and postoperative drug therapy to reduce infections [21, 22]. Another study [23] recommended the following regimens for complications of sinus elevation according to the patient group: for patients without allergy to penicillin, a combination of amoxicillin/clavulanic acid 1 g thrice a day (TID) and metronidazole 500 mg TID per os for 7 to 10 days; and for patients allergic to penicillin, a regimen composed of levofloxacin 400 mg BID per os until 72 h to symptom remission. In our study, development of a postoperative infection or maxillary sinusitis (14 cases) manifested as nasal congestion, headache, pain, fever, redness, or pain that worsened with bending forward with or without purulent drainage [19, 24, 25] was treated with amoxicillin/clavulanic acid as the first-line drug. When there was no improvement of symptoms after taking amoxicillin/clavulanic acid for a week, metronidazole was added to the regimen as recommended, together with amoxicillin for an additional week. Radiographic examinations of these patients revealed maxillary sinus mucosal thickening, air-fluid levels, and radiographic signs, such as radiopacity. Additional medication was administered for approximately a week (range, 5 days to 4 weeks). Patients were informed about guidelines for basic maxillary postoperative care in addition to the antibiotics prescription. Mouth gargle with 0.1% chlorhexidine solution was also recommended. Ecchymosis along with mild bleeding (16 cases) and wound disruption (6 cases) were observed, but these symptoms showed no impact on the survival rate of implant.

An association between sinus perforation and graft dislodgement into the sinus with disruption of the normal sinus physiology has been previously described [1, 4, 19, 24, 26, 27]. A disrupted mucociliary apparatus function and loss of the biologic barrier, caused by perforation of the membrane, can increase the invasion of bacteria into the sinus and cause infection [14, 28]. This may explain the increased incidence of secondary infections in our study.

The use of decongestants that may widen the ostia and improve nasal ventilation has been recommended [23]. However, chronic use of decongestants beyond 3 to 5 days should be discouraged, as they may result in significant rebound hyperemia and rhinitis medicamentosa. Horak et al. [29] reported that cetirizine (second-generation antihistamine)/pseudoephedrine is effective in the management of nasal congestion. In this study, we prescribed pseudoephedrine for cases with nasal congestion or rhinorrhea.

Successful implant placement is dependent on the achievement and maintenance of osseointegration [30]. Early studies with implant treatment relied on the placement of implants followed by healing period of 3 to 6 months during which the implants were protected from externally applied forces [31–34]. With the development of new implant types, surface technology, and advanced knowledge about the physiology of osseointegration, the requirement for delayed restoration of dental implants has been challenged [35–38]. However, implants placed in augmented sinus are different from conventional implants. Therefore, the timing of the implant loading is important for survival of implant placed in augmented sinus. Lang et al. [39] suggested that timing of sinus elevation and implant placement in relation to implant survival is affected by time of implant loading. Their results showed that implants that were immediately loaded regardless of the timing of the sinus elevation showed greater failure rates than implants in augmented bone that received a delayed loading protocol or those that were loaded immediately in sites that did not require a bone augmentation procedure. In this study, we applied the load on implant at least 5 months after implant placement and there was no failure.

Failure of bone graft or implant, which is commonly found in patients with perforated maxillary sinuses, was considered for additional debridement and irrigation or as the failure of the implant itself within one year of loading [40]. The data on implant survival rates vary by author. Proussaefs et al. [14] reported that implant success rate was 69.5% in the perforated maxillary sinus and 100% in the intact one, respectively. In the study by Khoury et al. [12], implant survival rate was lower when the membrane was perforated. On the other hand, some authors [8, 41] reported that maxillary sinus perforation does not have a negative effect on the success rate of implants. Schwartz-Arad et al. [7] also suggested that a maxillary sinus perforation affects postoperative complications such as sinusitis, but not the success rate of the implant. In this retrospective study, the survival rate of the implants in the patients who received sinus elevation of the perforated sinus membrane and implant placement with bone graft was 100%.

The limitation of this study was that we included the implants that were not loaded. Potential limitations can arise from smoking or nonsmoking, differentiation of the sinus septa, or the systemic disease of the patients. Thus, for future studies, evaluation of the prognosis after loading and clinical outcomes by prognosis factor is necessary.

The results of our study suggested that the repair of a perforated sinus membrane using absorbable collagen membrane can be a safe and predictable procedure.

## Conclusions

Sinus elevation can be successfully performed and produce a good outcome even in cases of abruptly perforated sinus membrane, regardless of the location or the size of the perforation. Repair using resorbable collagen membrane has predictable results and is a reliable technique. Further studies with a follow-up period for a long-term survival rate of implants in the maxillary sinus with perforated sinus membrane and confirmation of the present results are needed.
